# Associations Between Physical Activity and Living Alone: Are There Differences by Gender?

**DOI:** 10.1093/geroni/igaa057.1601

**Published:** 2020-12-16

**Authors:** Jianjia Cheng, Emily Nicklett

**Affiliations:** 1 University of Michigan, Ann Arbor, Ann Arbor, Michigan, United States; 2 University of Michigan, Ann Arbor, Michigan, United States

## Abstract

Nearly one in three adults aged 65 and older live alone in the U.S. Despite this increasing trend, there remains little understanding regarding the relationship between living alone and physical activity (PA) in older adulthood. Is living alone a risk factor for lower levels of PA among older adults? The effects of living arrangements on health behaviors could differ by gender; however, findings from prior studies on this topic have been mixed or inconclusive. This is one of few studies to examine whether PA is associated with living alone (vs. living with others) and whether the association differs by gender using longitudinal data. Our data were drawn from 2006-2014 Psychosocial and Lifestyle Questionnaire of the Health and Retirement Study, a nationally representative sample of older adults in the U.S. (N=17371, mean age=75.4). PA was measured repeatedly using metabolic equivalents of task (MET) estimated values accounting for the vigor and frequency of self-reported PA (range 0-31). Using mixed-effects linear regression, we found that living alone was significantly associated with higher levels of PA (Coeff.: 0.41, p<0.001). When examined separately by gender, living alone was associated with significantly higher PA among women (Coeff.: 0.47, p<0.001) but not among men (Coeff.: 0.29, p=0.14) after controlling for marital status, other sociodemographic characteristics, and health-related indicators. Our study provides evidence of gendered differences in initiating and maintaining health behavior change in relation to living arrangements. Findings provide implications for the design of PA promotion programs and policies for older adults.

